# TiO_2_ Films Modified with Au Nanoclusters as Self-Cleaning Surfaces under Visible Light

**DOI:** 10.3390/nano8010030

**Published:** 2018-01-08

**Authors:** Ting-Wei Liao, Sammy W. Verbruggen, Nathalie Claes, Anupam Yadav, Didier Grandjean, Sara Bals, Peter Lievens

**Affiliations:** 1 Laboratory of Solid-State Physics and Magnetism, KU Leuven, Celestijnenlaan 200D, Box 2414, BE-3001 Leuven, Belgium; tingwei.liao@kuleuven.be (T.-W.L.); anupam.yadav@kuleuven.be (A.Y.); didier.grandjean@kuleuven.be (D.G.); peter.lievens@kuleuven.be (P.L.); 2 Sustainable Energy, Air & Water Technology (DuEL), University of Antwerp, Groenenborgerlaan 171, BE-2020 Antwerp, Belgium; 3 Center for Surface Chemistry and Catalysis, KU Leuven, Celestijnenlaan 200F, BE-3001 Leuven, Belgium; 4 Electron Microscopy for Materials Research (EMAT), University of Antwerp, Groenenborgerlaan 171, BE-2020 Antwerp, Belgium; nathalie.claes@uantwerpen.be (N.C.); sara.bals@uantwerpen.be (S.B.)

**Keywords:** photocatalysis, gold nanoclusters, plasmonics, cluster beam deposition, stearic acid, self-cleaning surfaces

## Abstract

In this study, we applied cluster beam deposition (CBD) as a new approach for fabricating efficient plasmon-based photocatalytic materials. Au nanoclusters (AuNCs) produced in the gas phase were deposited on TiO_2_ P25-coated silicon wafers with coverage ranging from 2 to 8 atomic monolayer (ML) equivalents. Scanning Electron Microscopy (SEM) images of the AuNCs modified TiO_2_ P25 films show that the surface is uniformly covered by the AuNCs that remain isolated at low coverage (2 ML, 4 ML) and aggregate at higher coverage (8 ML). A clear relationship between AuNCs coverage and photocatalytic activity towards stearic acid photo-oxidation was measured, both under ultraviolet and green light illumination. TiO_2_ P25 covered with 4 ML AuNCs showed the best stearic acid photo-oxidation performance under green light illumination (Formal Quantum Efficiency 1.6 × 10^−6^ over a period of 93 h). These results demonstrate the large potential of gas-phase AuNCs beam deposition technology for the fabrication of visible light active plasmonic photocatalysts.

## 1. Introduction

Various semiconductor-based photocatalysts, such as TiO_2_, ZnO, SrTiO_3_, Fe_2_O_3_, FeOOH, WO_3_, CdS and III–V compound semiconductors, have drawn considerable attention in recent years [1,2,3,4,5,6,7,8,9,10,11,12], due to their multiple promising applications in solar energy harvesting [13,14,15], water cleaning [16,17,18], anti-bacterial surfaces [19], organic pollutants removal [20] and self-cleaning surfaces [21] as new construction materials for the improvement of the urban environment. The main factors that determine the photocatalytic properties of semiconductor-based photocatalysts are band gap, position of conduction band and valence band, mobility of photo-generated charge carriers and material stability [22]. Although titanium dioxide (TiO_2_) is a good photocatalyst towards oxidation of organic compounds under ultraviolet (UV) light [23], its photocatalytic properties need to be improved significantly in the visible range, especially under green light that corresponds to the maximum output range of the sun’s total irradiance spectrum. Surface modification with metal nanoparticles improves the photoabsorption rate in the visible range by (amongst other phenomena) hot electron injection effects produced by their localized surface plasmon resonance (LSPR) [24,25,26].

Most of the metal nanoparticle-TiO_2_ composites nowadays are synthesized in solution by various wet chemistry synthesis protocols that generally include inorganic acid in the particle synthesis, toxic ligands to protect the metal particles and functional groups that stabilize the nanoparticles (NPs) on the TiO_2_ surface [14,27,28]. It has been shown that the performance of the NPs is highly affected by the chemical environment during the preparation processes. Therefore, the use of acids and ligands in wet chemistry synthesis methods can have a significant inference on the NPs [29]. Furthermore, by ensuring the NPs are only present at the surface can reduce the usage of the amount of noble metals. Cluster beam deposition (CBD) greatly benefits from producing clusters in a well-controlled noble gas environment and then soft landing them on supports while maintaining their preformed structure with excellent control over size, shape, and composition [30]. In order to produce clean and controllable photocatalytic systems with only desired materials, physical cluster beam deposition methods that produce size-selective nanoclusters or nanoparticles (NCs/NPs) in noble gas environments, free of unwanted ligands or functional groups, present a very attractive alternative.

In this study, we report on the significant improvement of the photocatalytic activity of TiO_2_ P25 films modified with AuNCs produced by the laser ablation cluster beam deposition technology, with equivalent coverage ranging from 0 ML to 8 ML (1 ML ~1.5 × 10^15^ atoms/cm², ~0.5 μg/cm²) towards stearic acid degradation under UV (365 nm) and green LED light (515 nm) irradiation. The photocatalytic efficiency of AuNCs modified TiO_2_ films with an equivalent cluster coverage of 4 ML was enhanced by a factor of 4 compared to pristine TiO_2_ P25 under green LED irradiation while no significant effect of AuNCs on the activity was noticed under UV exposure.

## 2. Results

### 2.1. Morphology of Au Nanoclusters Modified Films

**Figure 1:**
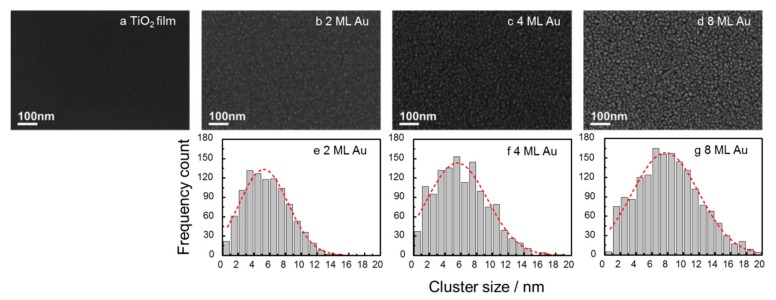
SEM images of Si wafers supported TiO_2_ P25 (**a**). Gas phase Au cluster modified TiO_2_ P25 films with 2 ML (**b**), 4 ML (**c**) and 8 ML (**d**) equivalent coverage as well as size distribution histograms of 2 ML (**e**), 4 ML (**f**) and 8 ML (**g**) samples. The red dash lines in the histograms are the fittings of cluster distributions.

Figure 1a shows a Scanning Electron Microscopy (SEM) image of the TiO_2_ P25 film, supported on a Si wafer. TiO_2_ P25 films are uniform and no apparent regions or island discontinuities are observed on the sample surface at this scale. SEM images of 2 ML, 4 ML and 8 ML of AuNCs modified TiO_2_ P25 films are presented in Figure 1b–d, respectively. Due to the lower ionization potential of Au compared to TiO_2_, the electron intensity from AuNCs is higher allowing AuNCs to be revealed. The cluster size is determined by the cluster projected area and the cluster size distributions are presented in Figure 1e (2 ML), Figure 1f (4 ML) and Figure 1g (8 ML). All the coated TiO_2_ P25 films are homogeneously covered by AuNCs. At the lowest coverage (2 ML) most of AuNCs have an average size of 5.2 nm and appear individually without any sign of aggregation while at the highest coverage (8 ML) AuNCs with a larger size of 7.8 nm and irregular shapes are observed suggesting a significant level of cluster coalescence. The morphology of the 4 ML sample consists mainly of individual clusters with an average size of 5.5 nm very similar to the NCs in the 2 ML sample. The mobility of the Au atoms on TiO_2_ has been studied by Scanning Tunneling Microscopy (STM) and the binding energy of Au atoms on TiO_2_ was investigated by Density Functional Theory (DFT). Both of these studies show that the binding energy of Au atoms and clusters is low enough so that Au clusters can become mobile on the TiO_2_ surface [31,32]. Therefore, when the coverage of Au clusters increases, the chance of Au clusters to coalesce will increase and result in larger and irregularly shaped particles.

**Figure 2:**
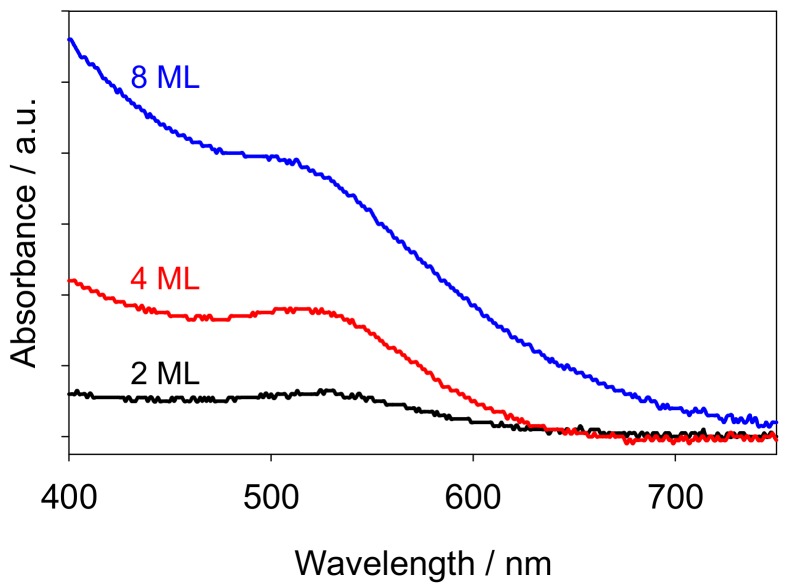
UV-Vis absorption spectra of 2 ML (black), 4 ML (red) and 8 ML (blue) AuNCs on TiO_2_ films, with a pristine TiO_2_ film as the background sample.

Optical characterization of the AuNCs revealed that for all three modified samples, a typical gold surface plasmon resonance (SPR) band is observed at a wavelength of ca. 520 nm, as expected for small gold clusters (Figure 2). The absorbance intensity of the SPR band increases as the gold loading on the sample increases. It is also striking that the absorption onset for the 8 ML sample is strongly red-shifted compared to the other samples and the SPR band is broadened. This is in full agreement with the larger average particle size, degree of coalescence and irregular shape of the particles observed by SEM for this particular sample.

### 2.2. Photocatalysis on Stearic Acid

**Figure 3:**
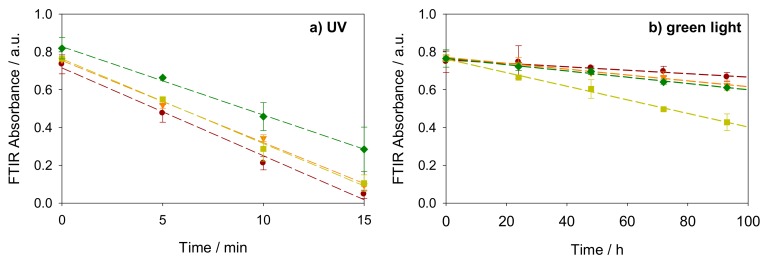
Evolution of integrated FTIR absorbance of stearic acid between 2800 to 3000 cm^−1^ as a function of illumination time on the sample of pristine TiO_2_ P25 film (red **●**) and Au cluster modified TiO_2_ P25 films with 2 ML (orange ▼), 4 ML (yellow ■) and 8 ML (green ♦) Au coverages under (**a**) UV and (**b**) green light illumination.

The integrated area of the Fourier-transform infrared (FTIR) absorbance spectra between 2800 and 3000 cm^−1^ as a function of time with different coverage is shown in Figure 3a,b for stearic acid degradation under UV and green LED light, respectively. The quantity of stearic acid remaining on the sample surface decreased linearly with time, indicating a zero order degradation kinetics. This is consistent with the self-cleaning behavior of plasmonic photocatalysts reported in earlier work [24]. A series of blank reference experiments was done in our previous work to exclude the possible contributions of photolysis, plasmonic heating or direct plasmonic catalysis on the noble metal clusters by investigating a blanc silicon wafer and samples with direct deposition of noble metal clusters but without TiO_2_. The results showed that these samples present no activity under either UV, 490 nm LED light, nor simulated solar light [24].

In Figure 3 red lines correspond to the TiO_2_ P25 films without Au cluster modification while the orange, yellow and the green lines to TiO_2_ P25 films modified with 2 ML, 4 ML, and 8 ML of AuNCs, respectively. The photocatalytic activities of TiO_2_ P25 under UV towards stearic acid degradation were not improved upon modification with AuNCs. The activity was even disrupted to some extent at the highest coverage of 8 ML. Under green LED light illumination, however, all the photocatalytic activities of AuNCs modified TiO_2_ films were enhanced significantly. These results are preferably expressed as “Formal Quantum Efficiency” (*FQE*) for comparison [33], which is defined as

$$FQE=\frac{\mathrm{the}\;\mathrm{rate}\;\mathrm{of}\;\mathrm{stearic}\;\mathrm{acid}\;\mathrm{degradation}\;\mathrm{in}\;\mathrm{molecules}/{\mathrm{cm}}^{2}/s}{\mathrm{the}\;\mathrm{rate}\;\mathrm{of}\;\mathrm{incident}\;\mathrm{light}\;\mathrm{in}\;\mathrm{photons}/{\mathrm{cm}}^{2}/s}.$$

**Figure 4:**
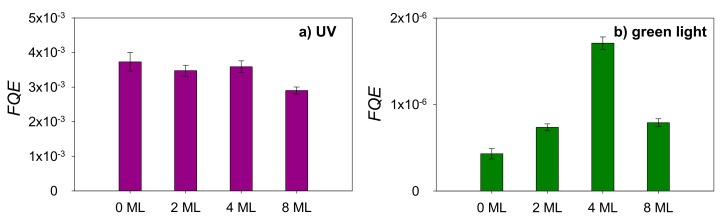
*FQE* under (**a**) UV and (**b**) green light illumination as a function of Au cluster coverage on TiO_2_ P25.

The *FQE* under UV and green light illumination as a function of Au coverage are presented in Figure 4a,b, respectively.

Under UV illumination, the performance of all samples is similar. The *FQE* of the pristine TiO_2_ P25 reference film under UV was measured to be 3.7 × 10^−3^. The value measured by Mills et al. was 4.8 × 10^−3^ for the same type of catalyst, which is in very good agreement with our result given the fact that their film was prepared with a three-time dipping-drying process that leaves a larger amount of catalyst on the film [33]. This result is also in line with the findings of Allain et al. for a mesoporous TiO_2_ film (1.02 × 10^−3^) [34]. Our results show that modification with noble metal NCs did not improve the activity of the pristine reference sample under UV irradiation nor disrupt it until an equivalent Au coverage of 8 ML is reached. For the latter 8 ML sample a 20% decrease in degradation efficiency is observed under UV illumination. This may be attributed to an incremental blocking of the catalyst active sites, or to the fact that a larger fraction of incoming light is prevented from reaching the catalyst in comparison to the samples featuring lower Au coverages.

Under pure green visible LED light, a very clear positive effect in the presence of metallic AuNCs for all the samples is observed. This is ascribed to the plasmonic effect of AuNCs, active in this wavelength range. At first, it should be noted that the pristine TiO_2_ P25 features a small photocatalytic activity under green LED light illumination, which is rather unexpected and controversial. In principle visible light cannot initiate photocatalytic reactions on a TiO_2_ surface, as its photon energy is much lower than the TiO_2_ P25 band gap of 3.1 eV [35]. A possible explanation could be related to the presence of defect states present in the band gap of TiO_2_ P25 that enable the generation of charge carriers in pristine TiO_2_ P25 upon pure visible light excitation as has been demonstrated by means of Electron Paramagnetic Resonance (EPR) measurements [36]. This low basal activity under visible light was also observed in our previous study and could not be attributed to thermal degradation nor to pure photolysis processes [24].

## 3. Discussion

Upon modification of TiO_2_ with AuNCs, a significant increase of the photocatalytic efficiency under 515 nm green light is observed for all coverages. Although a modest increment is found for 2 ML AuNCs modified TiO_2_ P25 films, a *FQE* 4 times greater than the reference and 2.5 times greater than the 2 ML sample was measured in the 4 ML sample. However, further doubling the AuNC coverage to an equivalent of 8 ML resulted in a lowered efficiency, which may again be explained by an excessive blocking of the active sites and/or by unwanted light scattering. The hypothesis is supported by the observation of the SEM images. The size of the NCs at the surface of the 8 ML sample is larger compared to the other lower coverages, which is suggesting cluster aggregation and coalescence happened on the sample surface. This is also confirmed by broadening of the UV-Vis absorption spectrum of the clusters. Furthermore, the surface of the TiO_2_ P25 powder film is almost fully covered by AuNCs. The photocatalytic activity of pure noble metal clusters was studied in previous work and it was shown there is no photocatalytic nor photo-thermal activity of noble metal nanoparticles as such [24]. It was also studied that the active site is located at the metal-support site. The availability of both metal and support are necessary for the reaction to occur. Therefore, as the AuNCs coverage increases on the TiO_2_ surface, the accessibility of the AuNCs-TiO_2_ site reduces [37]. As a combined result of these effects, the photocatalytic activity of 8 ML Au cluster modified TiO_2_ P25 film is reduced.

**Figure 5:**
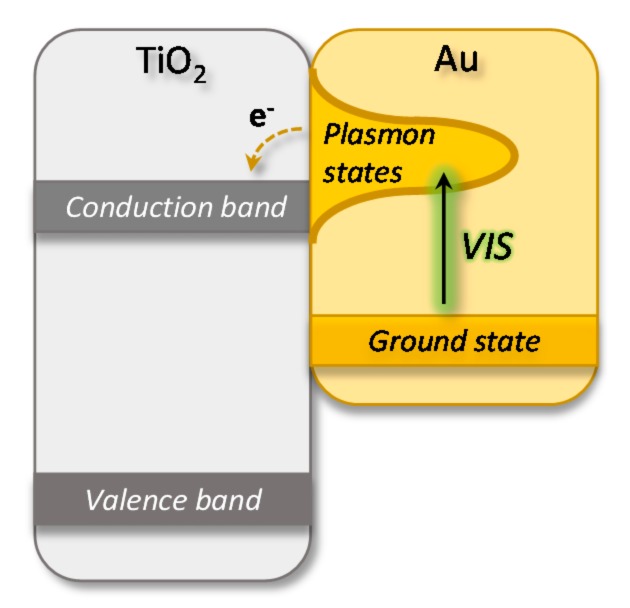
Schematic illustration of hot electron transfer from an excited plasmonic state on the gold nanoparticle, to the TiO_2_ conduction band. Modified from reference [20].

AuNCs feature a strong light absorption in the visible region where the absorbed energy can induce resonant oscillation of the free electrons present in the Au clusters [38]. The trapped energy can then be transferred to the coupled semiconductor substrate by a variety of mechanisms [39]. This was highlighted by electron paramagnetic resonance (EPR) studies of Au-TiO_2_ composites that showed that hot electron transfer from a gold excited plasmonic state into the TiO_2_ conduction band is an important underlying mechanism (Figure 5) [38,40].

One of the other advantages of the gas phase Au cluster deposition method, is that the active particles are exclusively located at the surface of the powder film where they can efficiently interact with incident photons, and not in the bulk. This increases the efficiency of material usage. For instance, the weight per area of 4 ML AuNCs is estimated to be only 2 μg/cm².

Although the IUPAC recommends to express the photocatalytic activity in terms of *FQE*, there is not yet a standard way to present the photocatalytic activity in the photocatalysis community. Therefore, it is difficult to compare the photocatalytic activity of our system to recent literature results. The 4 ML AuNCs film demonstrates higher photocatalytic activity compared to our previous studies on Au-Ag bimetallic plasmonic particles. It was found that TiO_2_ P90 (90% anatase, 10% rutile) modified with 1.5 wt % of Au_0.3_Ag_0.7_ plasmonic alloy nanoparticles under 490 nm teal light illumination resulted in a *FQE* of 0.39 × 10^−6^ [25]. In the present work, the *FQE* of 4 ML AuNCs modified TiO_2_ P25 under 515 nm green light is 1.7 × 10^−6^. In another study Au*_x_*Ag_1−*x*_ (*x* = 0.2 to 1) nanoparticles were deposited on TiO_2_ P25, resulting in a so-called rainbow photocatalyst that was 16% more effective than pristine TiO_2_ P25 under both simulated and real solar light toward stearic acid degradation (i.e., combined UV and visible light irradiation) [24]. The excellent photocatalytic activity enhancement observed under green light obtained with pure AuNCs in this study suggests that fabricating a similar “rainbow” photocatalyst using our physical gas phase cluster deposition method may lead to further enhancements of the photocatalytic activity, and is the subject of ongoing research.

## 4. Materials and Methods 

### 4.1. Preparation of Au Nanoclusters Modified Films

TiO_2_ P25 powder (Evonik, Hanau-Wolfgang, Germany, 70% of anatase and 30% of rutile) films were prepared by spin coating 1 wt % of titania powder ethanol suspension at 1500 rpm onto low-doped Si wafers with 3 cm in length and 1.5 cm in width followed by subsequent overnight drying at 70 °C. Next, TiO_2_ P25 powder films were modified with AuNCs with equivalent atomic coverages of 0 ML, 2 ML, 4 ML and 8 ML by depositing gas phase AuNCs produced with a laser ablation cluster source. Clusters are produced by pulsed laser (10 Hz, Nd:YAG lasers, Spectra Physics, Santa Clara, CA, USA, INDI) ablation of Au (ACI Alloy, San Jose, CA, USA, purity 99.995%) plate targets and condensation in a high pressure (9 bar) of inert gas (He, purity 99.9999%) [41]. The cluster source was cooled by liquid nitrogen, resulting in a NC temperature of about 100 K. In this study, the cluster size distribution was optimized for sizes in the order of 1000 atoms. Following a supersonic expansion into vacuum of the helium carried NC molecular beam, AuNCs were soft-landed (~500 m/s) on the P25 films in a ultra-high vacuum (UHV) cluster deposition chamber under a base pressure below 5 × 10^−10^ mbar [18]. The cluster flux was monitored by a quartz crystal microbalance (QCM) and the cluster coverage controlled by the deposition time under a fixed cluster flux.

### 4.2. Film Characterization

Secondary electron SEM images of TiO_2_ P25 films and AuNCs modified P25 films were acquired using a FEI Quanta 250 FEG environmental scanning electron microscope (Thermo Fisher Scientific—FEI, Hillsboro, OR, USA). The microscope was operated at 15 kV with a chamber pressure of 130 Pa. UV-Vis absorption spectra of the AuNCs were collected as-deposited on the films using a double beam UV2600 spectrophotometer (Shimadzu, Kyoto, Japan) equipped with film holders attached to a BaSO_4_ coated integrating sphere of 60 mm in diameter. A pristine TiO_2_ P25 film was used as the background sample.

### 4.3. Photocatalysis towards Stearic Acid Degradation

AuNCs modified films were then spin coated with 100 μL of a 0.25 wt % stearic acid solution in chloroform at 1000 rpm for 1 min and the stearic acid coated films dried in an oven at 70 °C for 15 min. Finally the samples were exposed to the ambient overnight to establish a thermal and humidity equilibrium before the photocatalytic measurements. The photocatalytic activity tests were performed at ambient conditions at a temperature of 32 ± 2 °C and a relative humidity of 34 ± 3% (monitored with a Humidity and Temperature Transmitter (Vaisala, Helsinki, Finland)) under UV and green light irradiation. The photon flux of UV and green light LED lamp emission were 2.9 × 10^15^ photons/cm^2^/s in 300 nm to 400 nm spectral range and 8.1 × 10^15^ photons/cm^2^/s in 450 nm to 600 nm spectral range, respectively, as measured with an Avantes Avaspec Spectroradiometer (Avantes BV, Apeldoorn, The Netherlands).

The degradation of stearic acid during illumination was measured by recording FTIR absorbance spectra (Thermo Nicolet 6700, Thermo Fisher Scientific, Madison, WI, USA) with 1 cm^−1^ resolution of the solid film placed at a fixed angle of 9° with the IR beam in order to minimize internal reflection effects. The quantity of stearic acid remaining on the sample surface was determined by integrating the area of the IR band between 2800 and 3000 cm^−1^, which consists of the asymmetric *ν_as_* (–CH_3_) vibration at 2958 cm^−1^, the asymmetric *ν_as_* (–CH_2_) at 2923 cm^−1^ and the symmetric *ν_s_* (–CH_2_) at 2853 cm^−1^ of the stearic acid hydro-carbon chain, and the results were plotted versus illumination time. The degradation experiments under UV illumination were performed over 15 min and the experiments under green light were monitored over a period of 93 h. This methodology was validated in earlier work of various authors [25,33,42].

## 5. Conclusions

We have demonstrated the excellent photocatalytic self-cleaning activity of a series of TiO_2_ P25 films modified with gas phase AuNCs synthesized by Cluster Beam Deposition. Photocatalytic stearic acid degradation is achieved under UV and green light illumination. At an atomic equivalent Au cluster coverage of 2 ML and 4 ML, the photocatalytic activities of the TiO_2_ films under UV are not influenced by the presence of the AuNCs; however, the photocatalytic activities of these two films under green LED light, especially for the sample with 4 ML coverage, are greatly improved. Increasing the AuNC coverage to 8 ML does not lead to any further improvement of the photocatalytic activity, as it even starts to disrupt the photocatalytic activity. Altogether, this work presents a versatile and valuable strategy for the fabrication of noble metal modified photocatalytic surfaces with a high control over the cluster size and coverage, without using aggressive solvents nor organic ligands to reduce the negative effect of these molecules.
